# Jianpi Bushen, a Traditional Chinese Medicine Therapy, Combined with Chemotherapy for Gastric Cancer Treatment: A Meta-Analysis of Randomized Controlled Trials

**DOI:** 10.1155/2018/4924279

**Published:** 2018-02-20

**Authors:** Yunbo Chen, Guijuan Zhang, Xiaoping Chen, Xuefeng Jiang, Fengjie Bie, Naijun Yuan, Yurong Wang, Xiaoqian Hao, Min Ma

**Affiliations:** ^1^College of Traditional Chinese Medicine, Jinan University, Guangzhou, Guangdong 510632, China; ^2^The First Affiliated Hospital, Jinan University, Guangzhou, Guangdong 510630, China; ^3^Wuning County People's Hospital, Jiujiang, Jiangxi 332300, China

## Abstract

*Objective. *To investigate the effects of Jianpi Bushen (JPBS), a traditional Chinese medicine that is used to invigorate the spleen and tonify the kidney, combined with chemotherapy for the treatment of gastric cancer.* Methods.* Literature retrieval was performed in PubMed, EMBASE, Cochrane Library, MEDLINE, CNKI, Wanfang Data Information Site, and VIP from inception to October 2017. Randomized controlled trials to evaluate the effects of JPBS combined with chemotherapy were identified. The primary reported outcomes were KPS (Karnofsky Performance Status), clinical curative efficiency, immune function, blood system, and nonhematologic system. Review Manager 5.3 (RevMan 5.3) was used for data analysis, and the quality of the studies was also appraised.* Results.* A total of 26 studies were included with 3098 individuals. The results of the meta-analysis indicated that treatment of gastric cancer with the combination of JPBS and chemotherapy resulted in better outcomes compared to chemotherapy alone.* Conclusion.* Evidence from the meta-analysis suggested that JPBS combined with chemotherapy has a positive effect on gastric cancer treatment. However, additional rigorously designed and large sample randomized controlled trials are required to confirm the efficacy and safety of this treatment.

## 1. Introduction

Gastric cancer is a significant contributor to the worldwide incidence of cancer and cancer-related deaths. The high incidence and mortality of gastric cancer make it one of the most deadly of cancers. The primary clinical treatment is surgery, which increases the survival rate of patients within five years after treatment [1].

Some reports have shown that gastric cancer patients that underwent resection or radical surgery subsequently suffered from residual tumor metastasis, malnutrition, and other complications, due to the effects of surgical trauma on the immune function, making surgery a less preferred option for patients with compromised immunity [1]. Another study reported that disease progression of tumor cells can be slowed down by the release of an immunosuppressive agent induced by postoperative tissue damage. Infection may interfere with the immune suppression function, not only affecting the patient's recovery, but also allowing residual cancer cells to proliferate, increasing the postoperative recurrence rate [2]. One study suggested that gastric cancer patients should be given early postoperative gastrointestinal nutrition intervention to improve gastrointestinal metabolism and autoimmune function, but further studies are required [3].

In China, the proportion of patients receiving adjuvant chemotherapy is about 60% [4]. Patients with early diagnosis who have undergone surgical treatment, preoperative or postoperative chemotherapy, and chemoradiotherapy can obtain good therapeutic effect. However, about 60% of patients are diagnosed with gastric cancer after the age of 65, and there is a significant risk of performing a surgery on these patients. Therefore, chemotherapy is widely used as a part of treatment of almost all patients with gastric cancer [5]. However, chemotherapy often causes adverse reactions in patients, including fatigue, anemia, vomiting, decreased neutrophils, thrombocytopenia, diarrhea, and nausea. The National Cancer Institute (NCI) reported that these chemotherapy regimens may produce severe to life-threatening effects (grade 3-4 adverse reactions according to the NCI's Common Toxicity Criteria) [5]. Additionally, drug resistance seriously limits the curative effect of chemotherapy.

In China, many clinical randomized controlled trials have demonstrated that Chinese herbal compounds can be beneficial as a part of adjuvant chemotherapy and can improve immunity, reduce adverse reactions, and reduce the possibility of cancer recurrence and metastasis [6, 7]. Chinese medicine (TCM) has now expanded outside of China and even Asia in the past 15 years and is now well received in Australia, Europe, and the United States. In the UK, about 2,000 shops and clinics provide Chinese medicine, including Chinese herbal medicine and acupuncture treatment. However, Chinese medicine treatment still lacks effective international evaluation [8].

Jianpi Bushen (JPBS) is a Chinese medicine mixture of several compounds designed to invigorate the spleen and kidney (this is the meaning of Jianpi Bushen). JPBS is widely used in combination with chemotherapy in China for the clinical treatment of gastric cancer. In this study, a meta-analysis is performed to evaluate the clinical efficacy and safety of JPBS used together with chemotherapy for gastric cancer treatment to determine whether it can improve the clinical efficacy of treatment of gastric cancer, enhance immune function, reverse drug resistance, and reduce adverse reactions.

## 2. Methods

### 2.1. Eligibility Criteria

*Types of Studies.* Our study included randomized controlled trials (RCTs) that evaluate the efficacy of JPBS combined with chemotherapy in the treatment of gastric cancer and other indicators, regardless of the length of treatment.

*Types of Participants.* Participants of any age and gender with a diagnosis of gastric cancer based on the “Guidelines for Standardized Diagnosis and Treatment of Gastric Cancer” (2013 edition) [34] were included. No subjects with significantly damaged liver and kidney function were included in the study.

*Types of Interventions.* Interventions were JPBS combined with chemotherapy for treatment of gastric cancer. Treatments that included other anticancer herbs were not included.

*Types of Outcome Measures. *The main outcome measures were Karnofsky Performance Status (KPS), clinical efficacy, blood system measurements (white cells, platelets, and hemoglobin), and immune parameters (CD3^+^, CD4^+^, CD8^+^, CD4/CD8^+^, NK^+^, E-rosette, and macrophages). The secondary indicators were other adverse reactions including gastrointestinal reactions, neurotoxicity adverse reactions, hand-foot syndrome, and bone marrow suppression.

Clinical efficacy was evaluated using the WHO evaluation criteria for the recent treatment of solid tumors [35]. Outcomes were complete remission (CR): all lesions disappeared and were maintained for four weeks; partial response (PR): the lesions were reduced by at least 30% and were maintained for 4 weeks; stable disease (SD): non-PR; and disease progression (PD): a 20% increase in the lesion or an increase of 5 mm in absolute value. CR and PR were considered effective treatments.

### 2.2. Literature Search

We performed literature retrieval electronically in the following databases: PubMed, EMBASE, MEDLINE, Cochrane Library, Chinese National Knowledge Infrastructure (CNKI), Wanfang Data Information Site, and Chinese Science and Technique Journals Database (VIP). All of the searches were conducted in October 2017 and included all articles in those databases prior to that time. The search terms used are as follows: (“Jianpi Bushen” OR “bushenjianpi” OR “Jianpi Bushen and chemotherapy” OR “bushenjianpi and chemotherapy”) AND (“gastric cancer” OR “cancer of the stomach” OR “gastric carcinoma”). These terms were translated into Chinese when searching the Chinese databases. In the process of screening the literature, we identified two articles describing studies with compound E Jiao Jiang (compound donkey-hide gelatin slurry, abbreviated as FEJ) combined with chemotherapy for gastric cancer. Because FEJ contains JPBS, these studies were also included in the meta-analysis.

### 2.3. Study Selection and Data Extraction

Two researchers (Xiaoqian Hao and Naijun Yuan) independently identified the relevant studies by reading the titles and abstracts and excluded documents that did not meet the inclusion criteria. The full text of the remaining studies was then read, and studies were assessed for inclusion in the meta-analysis based on the inclusion and exclusion criteria. To ensure accuracy and reliability, all the data and other clinical findings about the patients' characteristics, treatment details, and other clinical outcomes were extracted independently using standardized data collection tables from two investigators (Fengjie Bie and Yurong Wang). To avoid subjective bias, the author's name, the year and country of the paper published in the journal, and the titles were omitted in the data extraction. Two collaborators (Guijuan Zhang and Min Ma) jointly resolved disagreements about research content or data extraction. The other researchers (Xuefeng Jiang and Xiaoping Chen) independently extracted the data as follows: (1) the study design summary, including demographic characteristics, randomized methods, and implementation of blind methods, and (2) the sample size, short-term clinical effects, KPS scores, adverse reactions, and immunological expression in the treatment group and control group.

### 2.4. Risk of Bias in Individual Studies

We assessed the risk of bias of the included studies according to the Cochrane Handbook for Systematic Reviews of Interventions (Chapter 8.5; Higgins, 2011). This assessment included seven aspects: random sequence generation, allocation concealment, blinding of participants and investigators, blindness of outcome assessments, incomplete outcome data, selective outcome reporting, and other biases. We judged each aspect as having low, unclear, or high bias based on the Cochrane criteria.

### 2.5. Data Synthesis and Analysis

We used Review Manager 5.3 (RevMan 5.3) for data analysis. We analyzed the statistics by means of the mean difference (MD), with 95% confidence interval (CI). The heterogeneity of the included studies was assessed by *Q* and *I*^2^ test statistics. For *Q* statistics, a value of *P* < 0.05 was considered to have significant difference. We tested random effects models for meta-analysis when significant heterogeneity existed (*P* < 0.05 and *I*^2^ > 50%) among the included studies. Otherwise, fixed-effects models were applied. Funnel plots were used to evaluate publication bias when more than ten studies were identified.

## 3. Result

### 3.1. Description of Studies

**Figure 1:**
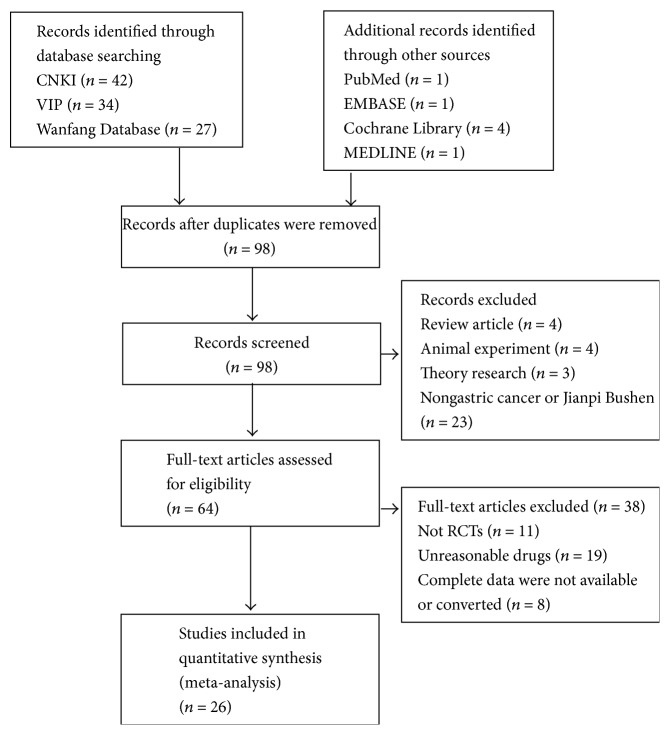
Study selection flow diagram.

We identified 178 potentially relevant articles. After screening titles and abstracts, 80 articles were excluded as nonclinical studies, expert experience, or case reports. We reviewed the remaining 64 studies in depth, and 38 studies were excluded because they did not meet our inclusion criteria, 11 of which were not RCTs, 19 articles reported treatment performed in combination with other traditional Chinese medicine therapies, and 4 were excluded because the outcome index did not meet the demand. Therefore, a total of 26 articles [9–33, 36] involving 3098 participants met our inclusion criteria. The screening process is summarized in a PRISMA flow diagram and presented in Figure 1. The 26 studies included 3098 participants: 1726 in the experimental group and 1372 in the control group. All studies were conducted in China. All studies included were two-group parallel designed studies. The detailed characteristics of the included studies are listed in Table 1.

### 3.2. Evaluation of the Clinical Efficacy

#### 3.2.1. Clinical Curative Efficiency

**Figure 2:**
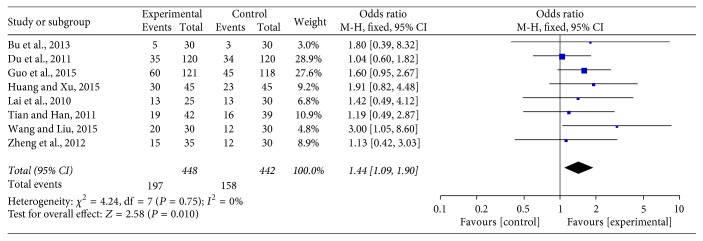
Forest plot of improved clinical curative efficiency.

Eight trials [15–17, 21–24, 31] with a total of 890 patients reported clinical curative efficiency. The heterogeneity test (Chi^2^ = 4.24, *P* = 0.75, *I*^2^ = 0%) indicated low statistical heterogeneity between studies. A fixed-effects model was applied to calculate the combined odds ratio (OR) and 95% CI as 1.44 (1.09, 1.90), *P* = 0.010, indicating a statistically significant difference between groups of JPBS combined with chemotherapy and chemotherapy alone. This indicates that JPBS combined with chemotherapy in the treatment of gastric cancer can significantly improve the efficiency of clinical curative effect when compared with chemotherapy alone (see Figure 2).

#### 3.2.2. KPS Score Evaluation

**Figure 3:**
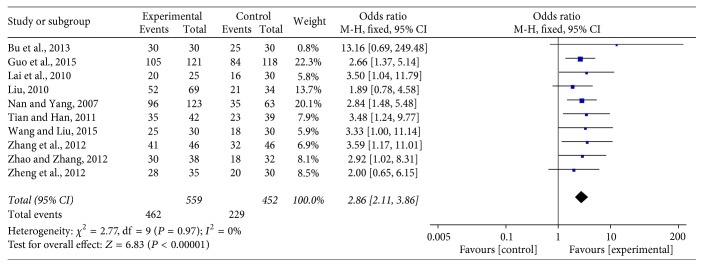
Forest plot of improved KPS.

Ten studies [13, 15–17, 21, 22, 24, 26, 29, 33] assessed KPS score in 1011 patients. The result showed that there was no statistical heterogeneity between studies (Chi^2^ = 2.77, *P* = 0.97, *I*^2^ = 0%), so a fixed-effects model was used to calculate the combined OR and 95% CI as 2.86 (2.11, 3.86), *P* < 0.00001. This indicates that there is a statistically significant difference between the two groups, showing that JPBS combined with chemotherapy may further increase KPS score to improve quality of life when compared with treatment of chemotherapy alone (see Figure 3).

#### 3.2.3. Immune Function (Figure 4)

The expression level of CD3^+^, a marker of immune function, was measured and reported in 6 of the 26 included trials [10, 12, 15, 21, 23, 24], containing 435 patients. The result of the heterogeneity test (Chi^2^ = 67.02, *P* < 0.00001, *I*^2^ = 93%) indicated statistically significant heterogeneity between studies. Results show mean difference (MD) = 8.13, 95% CI: 4.57 to 11.69, *P* < 0.00001, indicating a statistically significant difference between JPBS combined with chemotherapy group and chemotherapy group. These results show that JPBS combined with chemotherapy for the treatment of gastric cancer can increase CD3^+^ expression.

Six trials [10, 12, 15, 21, 23, 24], including 435 patients, reported CD4^+^ expression level. The heterogeneity test showed Chi^2^ = 27.89, *P* < 0.0001, and *I*^2^ = 82% in the meta-analysis, indicating statistically significant heterogeneity between studies. Based on the heterogeneity test, the MD and 95% CI were calculated as 4.79 (2.83, 6.75), *P* < 0.00001, indicating a statistically significant difference between the two groups. This result shows that JPBS combined with chemotherapy for the treatment of gastric cancer can significantly improve the CD4^+^ expression level.

Six trials [10, 12, 15, 21, 23, 24] with 435 cases reported CD8^+^ expression level. There was statistical heterogeneity between studies as evaluated by the heterogeneity test (Chi^2^ = 40.93, *P* < 0.00001, *I*^2^ = 88%). The MD and 95% CI were −4.26 (−7.03, −1.50), *P* = 0.002, indicating that JPBS combined with chemotherapy for the treatment of gastric cancer does not improve the CD8^+^ expression level.

The expression of CD4^+^/CD8^+^ was also reported by the same 6 trials [10, 12, 15, 21, 23, 24], including 435 patients. The heterogeneity test showed Chi^2^ =18.00, *P* = 0.003, and *I*^2^ = 72%, indicating large statistical heterogeneity between studies. The MD and 95% CI were 0.33 (0.23, 0.43), *P* < 0.00001, indicating a statistically significant difference between the two groups. This shows that JPBS combined with chemotherapy can significantly improve the expression level of CD4^+^/CD8^+^ for the treatment of gastric cancer.

NK^+^ levels were reported in six trials [10, 12, 21, 23, 27, 36] with 356 patients. The MD and 95% CI were 2.41 (1.43, 3.39), *P* < 0.00001, indicating that JPBS combined with chemotherapy for treatment of gastric cancer can significantly improve the NK^+^ expression level.

Three studies [27, 29, 36] with 442 patients applied E-rosette as an outcome measure. The heterogeneity test showed Chi^2^ =257.77, *P* < 0.00001, and *I*^2^ = 99%, indicating large statistical heterogeneity between studies. The MD and 95% CI were 2.10 (−1.85, 6.04), *P* = 0.30, indicating that JPBS combined with chemotherapy did not affect E-rosette expression.

Finally, data were extracted from two studies [29, 36] including 326 patients to assess macrophage expression levels. The heterogeneity test showed Chi^2^ =20.25, *P* < 0.00001, and *I*^2^ = 95%, indicating large statistical heterogeneity between studies. The MD and 95% CI were 2.53 (−0.02,5.08), *P* = 0.05. This indicates that JPBS combined with chemotherapy for the treatment of gastric cancer significantly increased the macrophage expression level.

### 3.3. Safety Evaluation

#### 3.3.1. Safety Evaluation of the Blood System (Figure 5)

Fifteen trials [9, 12, 13, 15, 17, 19, 20, 22, 24, 25, 27, 29, 30, 33, 36] with 2218 patients reported the decrease of white blood cells (WBC) occurrence rate. The meta-analysis showed that the OR and 95% CI were 0.21 (0.16,0.26), *P* < 0.00001, showing a statistically significant difference between the two treatment groups. This indicates that JPBS combined with chemotherapy can significantly reduce the rate of WBC decline compared to chemotherapy alone for the treatment of gastric cancer.

The rate of platelet (PLT) decrease was reported by nine studies [12, 15, 17, 19, 20, 22, 27, 29, 33] with 1173 cases. In the meta-analysis, the OR and 95% CI were 0.30 (0.19, 0.48), *P* < 0.00001, indicating a statistical difference between the two treatment groups. This indicates that JPBS combined with chemotherapy can greatly reduce the rate of platelets decline when used in the treatment of gastric cancer when compared to chemotherapy alone.

Seven trials [11, 12, 15, 17, 19, 24, 33] including 648 patients reported the levels of hemoglobin (Hb). The OR and 95% CI were 0.33 (0.19,0.59), *P* = 0.0002, indicating a statistically significant difference between the two treatment groups. This result suggests that JPBS combined with chemotherapy can greatly reduce the rate of hemoglobin decline during gastric cancer treatment when compared with chemotherapy alone.

#### 3.3.2. Nonhematologic Safety Evaluation (Figure 6)

The change of gastrointestinal reaction was reported by twelve trials [12, 15, 17, 19, 20, 22, 24, 25, 27–29, 36] with 1919 patients. The OR and 95% CI were 0.31 (0.24,0.40), *P* < 0.00001, indicating a statistical difference between the two treatment groups and suggesting that JPBS combined with chemotherapy for the treatment of gastric cancer can reduce the incidence of gastrointestinal reaction when compared with chemotherapy alone.

Five trials [12, 15, 21, 22, 26] that included 356 cases reported the incidence of neurotoxicity adverse reaction. The OR and 95% CI were 0.33 (0.20,0.55), *P* < 0.0001, indicating a statistical difference between the two treatment groups and suggesting that JPBS combined with chemotherapy for the treatment of gastric cancer can greatly reduce neurotoxicity adverse reactions when compared with chemotherapy alone.

Five trials [15, 17, 21, 22, 24] including 495 cases reported changes in hand-foot syndrome. The OR and 95% CI were 0.31 (0.21,0.45), *P* < 0.00001, indicating a statistical difference between the two treatment groups and suggesting that JPBS combined with chemotherapy for the treatment of gastric cancer can greatly reduce the damaging incidence of hand-foot syndrome when compared with chemotherapy alone.

The incidence of myelosuppression was reported by three studies [14, 21, 22] including 196 cases. In the meta-analysis, the OR and 95% CI were 0.31 (0.17,0.56), *P* = 0.0001, indicating a statistical difference between the two treatment groups. This result indicates that JPBS combined with chemotherapy can greatly reduce the incidence of myelosuppression, compared with chemotherapy alone, when used in the treatment of gastric cancer.

### 3.4. Risk of Bias

**Figure 7:**
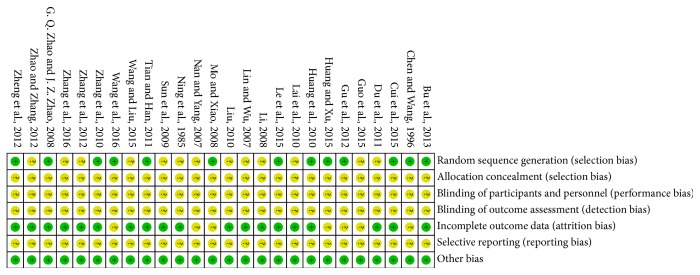
Risk of bias summary of included studies.

**Figure 8:**
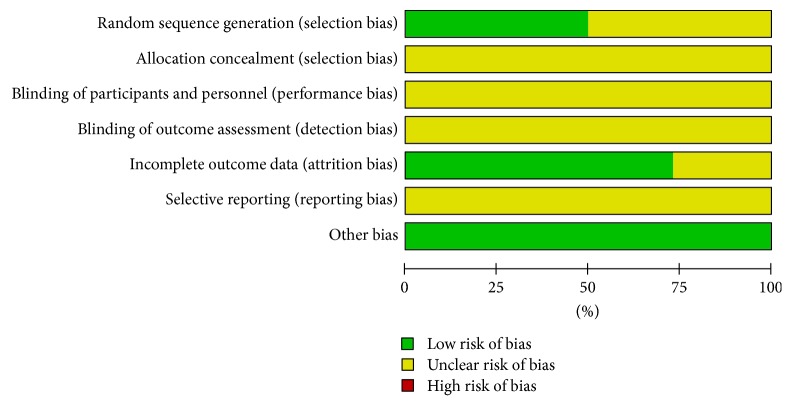
Risk of bias graph: review authors' judgments about each risk of bias item presented as percentages for all included studies.

We utilized the guidelines of the Cochrane Handbook for Systematic Reviews of Interventions (Chapter 8.5; Higgins, 2011) to evaluate the risk of bias for each included article. The included studies all claimed randomization, but the methods used for random sequence generation were reported by only 13 of the 26 trials [9, 10, 12, 16, 18, 19, 21, 23–25, 28, 30, 32]. None of the studies mentioned allocation concealment or described the process of blinding of participants and personnel and blinded outcome assessment, 26 of which were reported to have unclear risk of bias. Seven studies [9, 17, 19, 23, 28, 29, 32] did not provide all required information and the details of the data. Since study protocols were not available, selective reporting was identified as an unclear risk in all included studies (see Figures 7 and 8).

### 3.5. Publication Bias Analysis

**Figure 9:**
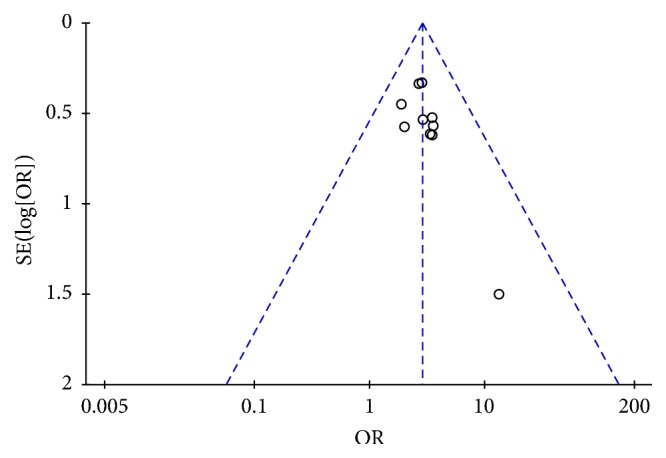
Funnel plot of KPS score evaluation.

**Figure 10:**
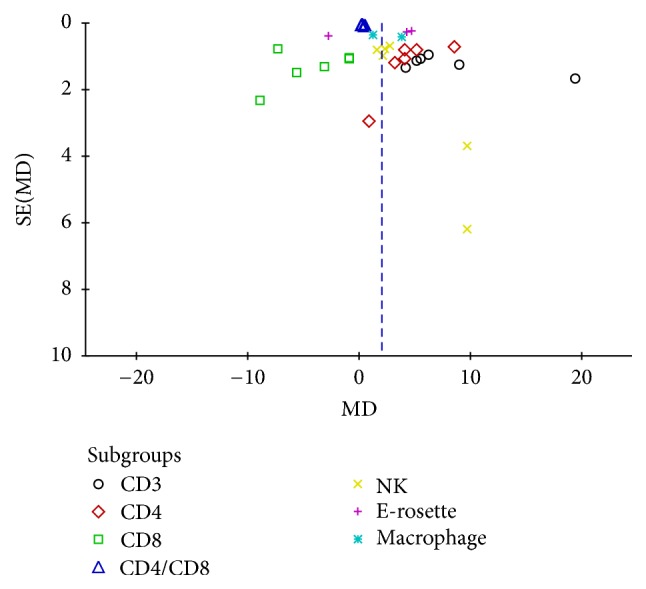
Funnel plot of immune function.

**Figure 11:**
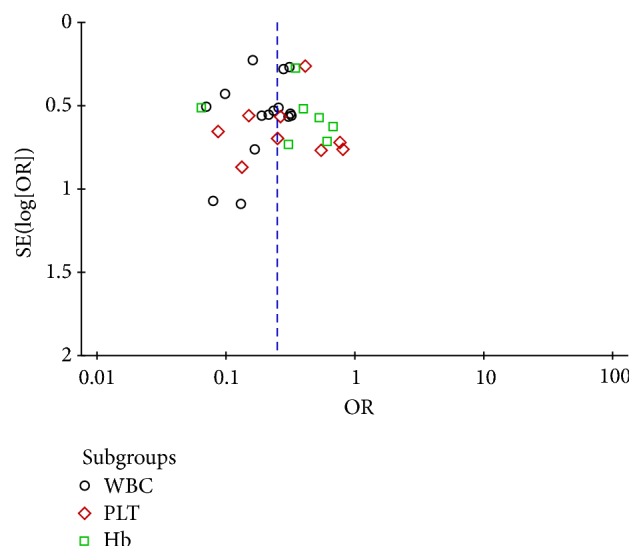
Funnel plot of safety evaluation of the blood system.

**Figure 12:**
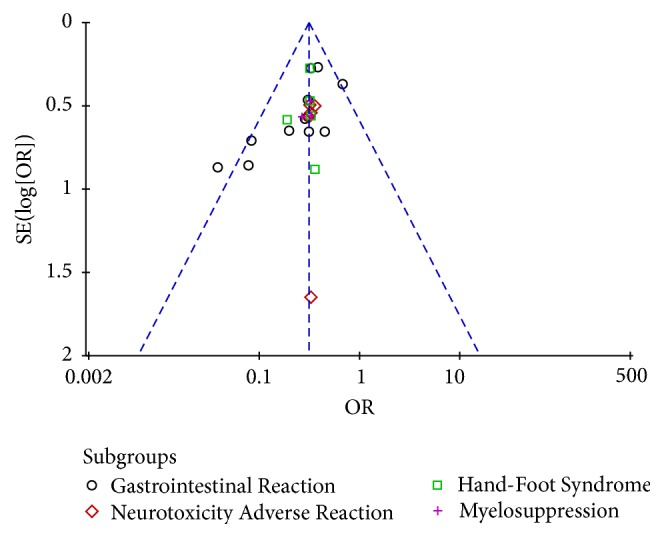
Funnel plot of nonhematologic safety evaluation.

Figures 9–12 present the funnel plots generated for studies with data on KPS score, immune function, safety evaluation of the blood system, and nonhematologic safety evaluation. The results showed that all points in the funnel plots were asymmetrical, indicating that there may have been publication bias in our study that might influence the results of our analysis.

### 3.6. Composition of JPBS

Table 2 lists the traditional composition of JPBS. The basic composition is dangshen (*Codonopsis pilosula*), baizhu (*Atractylodes*), fuling (*Poria cocos*), huangqi (*Astragalus membranaceus*), gancao (licorice), danggui (*Angelica*), chenpi (dried tangerine peel), banxia (*Pinellia ternata*), baishao (Radix Paeoniae Alba), shanzha (hawthorn), jineijin (Endothelium Corneum Gigeriae Galli), gouqi (Chinese wolfberry), nvzhenzi (Fructus Ligustri Lucidi), buguzhi (psoralen), tusizi (the seed of Chinese dodder), yiyiren (Semen Coicis), ejiao (donkey-hide gelatin), shudihuang (prepared* Rehmannia* root), shanzhuyu (Fructus Corni), jixueteng (Lignum Millettiae), and huangjing (sealwort) [9–33, 36]. Of these components, dangshen, baizhu, fuling, huangqi, gancao, danggui, chenpi, banxia, baishao, shanzha, and jineijin are included to strengthen the spleen and replenish qi. Gouqi, nvzhenzi, buguzhi, tusizi, yiyiren, ejiao, shudihuang, shanzhuyu, jixueteng, and huangjing act to tonify the kidney. In Chinese medicine, these herbs together invigorate the spleen and kidney and regulate yin and yang.

## 4. Discussion

### 4.1. Summary of Main Results

According to Chinese medicine, spleen and kidney deficiency is the basis of the incidence of gastric cancer [33], and further dysfunction of organs such as the liver and stomach, qi stagnation, blood stasis, and phlegm agglutination eventually lead to the occurrence of tumors. Thus, the main principle of the Chinese medicine treatment for gastric cancer is to invigorate the spleen and kidney (this is the meaning of Jianpi Bushen) [33]. We performed a meta-analysis of data that support the efficacy of this treatment strategy. As an auxiliary therapy for gastric cancer, JPBS combined with chemotherapy improved the efficiency of clinical curative effect, increased KPS score, increased the levels of CD3^+^, CD4^+^, CD4^+^/CD8^+^, NK^+^, and macrophages, and reduced the level of CD8^+^ and the rates of decline of WBC, PLT, and Hb. Additionally, patients who received JPBS combined with chemotherapy showed reduced incidence of gastrointestinal reaction, reduced neurotoxicity adverse reaction, reduced hand-foot syndrome, and reduced incidence of myelosuppression.

### 4.2. Analysis of JPBS Formulation

Many studies have shown that the drugs contained in JPBS show antitumor properties and can improve immune function [37].* Astragalus* contains polysaccharides that can stimulate the production of TNF-a (Tumor Necrosis Factor) by macrophages, alter levels of NO, increase expression of cytokines, and promote the proliferation of T-cells [38]. Another study showed that the active ingredients of* Astragalus* (huangqi) mucosal immune function can improve and enhance the killing ability of NK cells [39].* Angelica* (danggui) affects the immune function, can block the phagocytosis of macrophages, and can reduce TNF-a secretion [40].* Codonopsis* (dangshen) can improve NK cell killing activity, increase T-cell level, and promote immune function via the unique biological activity of the* Codonopsis* polysaccharide [41].* Atractylodes* (baizhu) polysaccharide can stimulate mice to produce specific IgG antibodies and nonspecific IgG antibodies (cross-antibody), thus promoting immunity [42].

This meta-analysis suggested that JPBS intervention indeed improves the clinical effect and the quality of survival (KPS) and strengthens the immune function (CD3+, CD4^+^, CD8^+^, CD4^+^/CD8^+^, NK^+^, and macrophages). Additionally, JPBS reduced the adverse effects of chemotherapy such as blood toxicity (WBC, PLT, and Hb effects), gastrointestinal reaction, neurotoxicity adverse reaction, hand-foot syndrome, and bone marrow suppression. However, little effects on E-rosette were seen, possibly due to the small sample size. Future large-scale studies can address the details of these effects more comprehensively.

Overall, this analysis revealed the effectiveness and safety of the use of the traditional Chinese medicine Jianpi Bushen combined with chemotherapy for the treatment of gastric cancer. The effects are striking, and these results should serve as the scientific basis for worldwide use of this powerful treatment.

### 4.3. Limitations of Research

There are some limitations of this study that preclude us from reaching definite conclusions. First, according to the statement published by the members of the International Committee of Medical Journal Editors in September 2004, all clinical trials are required to be registered in a clinical trial registry before enrolling subjects in the study [43]. This registration should be described in the publication. However, none of the included studies was registered. Second, the methodological quality of the included RCTs was generally low. Most of them do not describe allocation concealment and blinding, which limit the credibility of the results. Publication bias may be present. Third, high clinical heterogeneity could lower the reliability and validity of the research results. Fourth, most of these included studies were published in Chinese journals, limiting the potential extrapolation of the results. Finally, the search strategy may not identify all relevant studies. Given these limitations, additional well-controlled large studies are required to confirm these findings.

## 5. Conclusion

Traditional Chinese medicine Jianpi Bushen therapy combined with chemotherapy in the treatment of gastric cancer may really enhance the immunity of patients to improve the clinical efficacy and safety. However, the detailed mechanism of how JPBS works together with chemotherapy remains unclear and the quality of the included studies was relatively inadequate. Hence, it is necessary to carry out more high-quality, large sample, multicenter, prospective, randomized, double-blind clinical trials to further evaluate the efficacy of JPBS and chemotherapy treatment for gastric cancer.

## Figures and Tables

**Figure 4:**
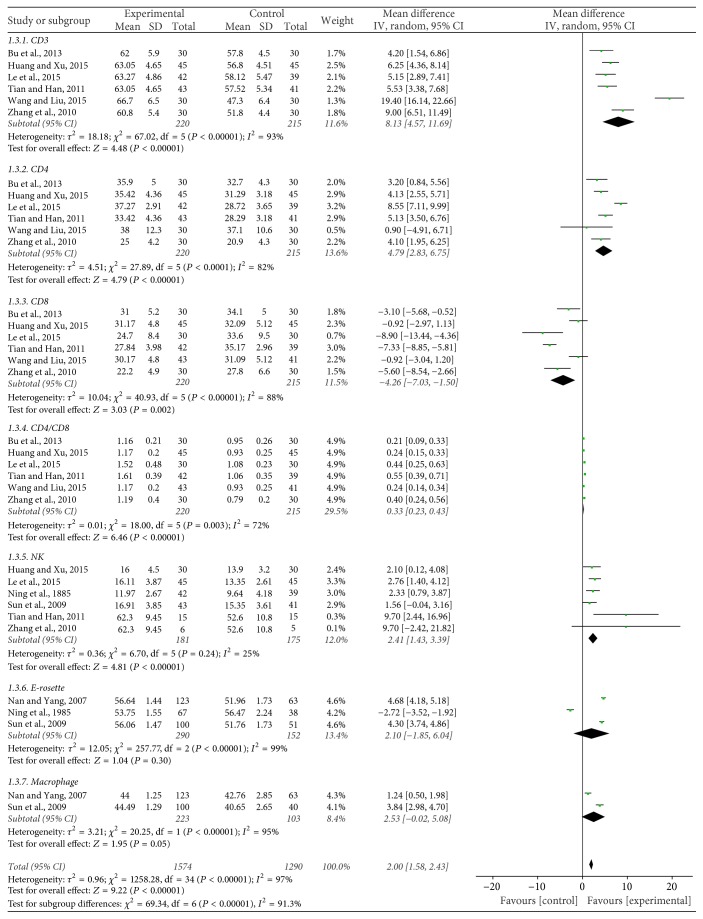
Forest plot of immune function.

**Figure 5:**
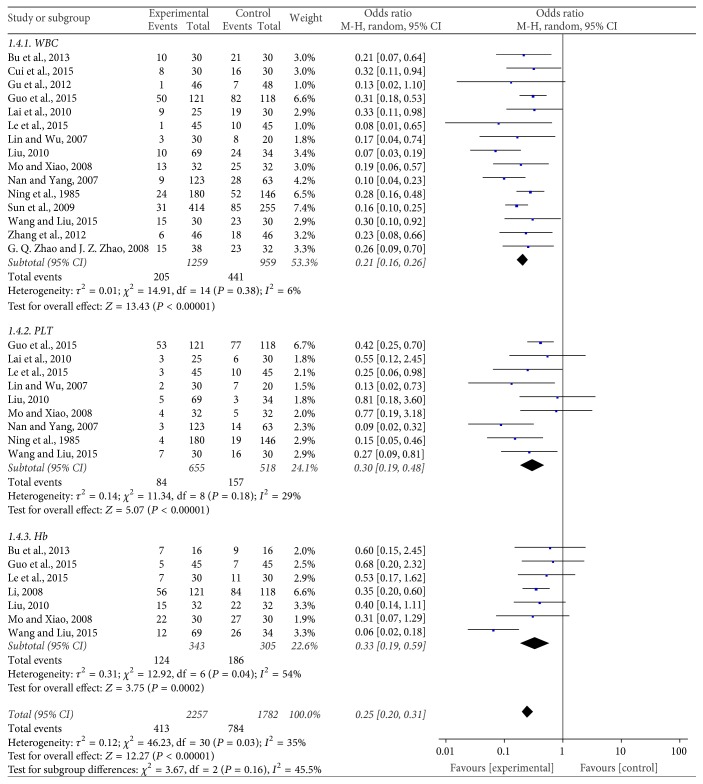
Forest plot of the blood system.

**Figure 6:**
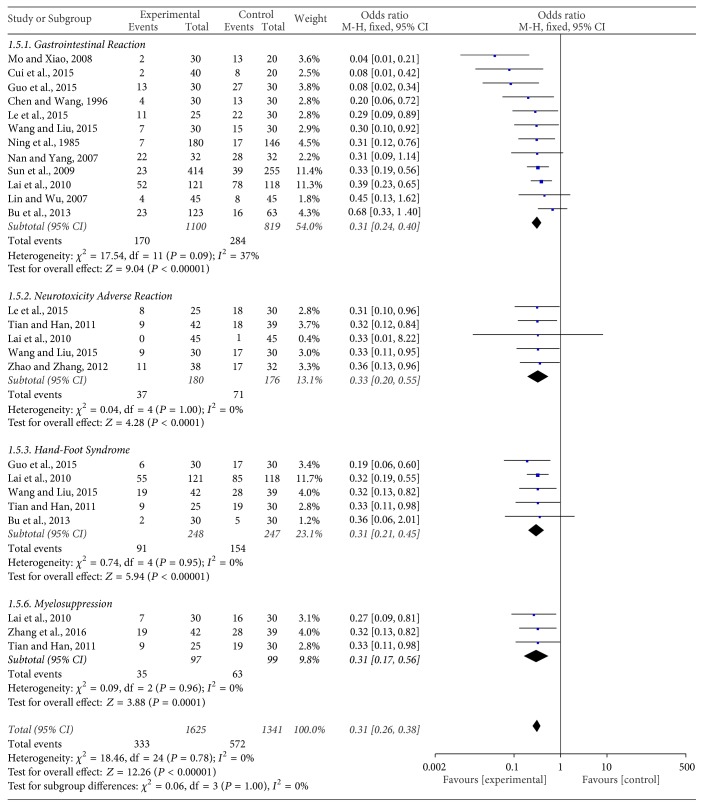
Forest plot of the nonhematologic system.

**Table 1:** Characteristics of included studies.

Included trials	Sample size (T/C)		Age (T/C)		Cancer stage	Intervention (T/C)^a^	Treatment course (C/W/D)	KPS
Gu et al., 2012	46	48	51.7	58.4	-	FEJ + TCF, DC, FOLFOX4, DCF	1 C, 4 W/C	>60
Nan and Yang, 2007	130	86	18–75		III-IV	BJD + MFV	1 C, 6 W/C	≥60
Zhang et al., 2010	30	30	45.2 ± 12.1	46.3 ± 11.8	-	BJD + PTX, OXA, CF, 5-FU	2–4 C, 14 D/C	-
Li, 2008	16	16	53.2	53.6	-	JBD + TF	2 C, 3 W/C	≥70
G. Q. Zhao and J. Z. Zhao, 2008	38	32	59.1 ± 9.4	57.8 ± 8.2	I–III	JBD + MFP	4 C, 28 D/C	-
Le et al., 2015	45	45	62.5 ± 5.5	61.7 ± 5.8	II–IV	JBD + FOLFOX4	4 C, 14 D/C	≥60
Zhang et al., 2012	46	46	45.35 ± 6.7	48.35 ± 7.2	I–IV	JBD + PTX, CF, 5-FU	4 C, 2 W/C	≥60
Zhang et al., 2016	30	30	48.52 ± 4.58	45.52 ± 5.58	II-III	JBD + FOLFOX4	6 C, 1 W/C	-
Du et al., 2011	120	120	55.2		IV	JBD + FOLFOX4	2 C, 21 D/C	≥60
Wang and Liu, 2015	30	30	70.2 ± 2.3	72.6 ± 2.1	III-IV	JBD + EPI, DDP, CAP	4 C, 10 D/C	≥60
Zheng et al., 2012	35	30	64	63	IIIB-IV	JBD + L-OHP, 5-FU/CF	2 C, 21 D/C	≥60
Guo et al., 2015	121	118	73.1 ± 4.4	74.1 ± 4.0	III-IV	JBD + XELOX	6 C, 3 W/C	≥60
Huang et al., 2010	20	20	46.7 ± 11.9	45.3 ± 12.5	-	JBD + 5-FU, CF, DDP/PTX	2–4 C, 2 W/C	-
Mo and Xiao, 2008	32	32	47.5 ± 8.6	48.3 ± 7.9	I–IV	JBD + FLP	4 C, 28 D/C	>60
Lin and Wu, 2007	30	20	24~65		-	JBD + PTX, CF, FU	4 C, 2 W/C	-
Tian and Han, 2011	42	39	56.83 ± 8.74	55.72 ± 7.32	-	JBD + DOC, DDP	2 C, 21 D/C	≥60
Lai et al., 2010	25	30	44	48	-	JBD + TAX, 5-FU, CF	4 C, 10 D/C	≥50
Huang and Xu, 2015	45	45	62.5 ± 5.5	61.7 ± 5.8	-	JBD + tegafur	1 C, 4 W/C	≥60
Sun et al., 2009	414	255	-		-	JBD + CTX	6 C, 4 W/C	-
Bu et al., 2013	30	30	48	49	III A-IV	FEJ + XELOX	3 C, 3 W/C	≥60
Cui et al., 2015	30	30	61	57.5	-	JBD + TAX, DDP	6 C, 3 W/C	-
Zhao and Zhang, 2012	38	32	69.7	71.3	III-IV	JBD + L-OHP, 5-FU	3 C, 2 W/C	>50
Ning et al., 1985	180	146	51		III-IV	JBD + MMC, 5-FU, VCR	1 C, 6 W/C	-
Wang et al., 2016	40	38	32–75	35–73	II-IIIA	JBD + FOLFOX4	12 C, 4 W/C	≥80
Liu, 2010	69	34	25–84	32–82	II-III	JBD + FOLFOX4	6–8 C, 2 W/C	>60
Chen and Wang, 1996	40	20	58	60	II-III	JBD + 5-FU, MMC, ADM	1 C, 6 W/C	-

*Note.* T/C: treatment group/control group; C: cycle; W: week; D: day; KPS: Karnofsky; FEJ: Compound E Jiao Jiang; TCF: PTX (paclitaxel) and DDP (cisplatin) and 5-Fu (5-fluorouracil); DC: DOC (docetaxel) and DDP; FOLFOX4: OXA (oxaliplatin) and CF (calcium folinate) and 5-Fu; DCF: DOC and DDP and 5-Fu; BJOL: bushenjianpi oral liquid; MFV: MMC (mitomycin) and 5-Fu and VCR (vincristine); BJD: bushenjianpi decoction; JBD: Jianpi Bushen decoction; TF: PTX and 5-Fu; MFP: 5-Fu and MMC and DDP and CF; EPI: pirarubicin; CAP: capecitabine; L-OHP: oxaliplatin; XELOX: OXA and CAP; FLP: CF and 5-Fu and DDP; FU: fluorouracil; CTX: cytoxan; TAX: paclitaxel; VCR: leurocristine; ADM: doxorubicin. ^a^The treatment group was given JPBS Chinese medicine combined with chemotherapy, and the control group was given only chemotherapy.

**Table 2:** Chinese medicine composition table.

JPBS Chinese herbal medicine components (sorted by frequency)	Daily dose
Chinese medicine for invigorating the spleen	
Dangshen [9–28]	0.01~0.03 kg
Baizhu [11–17, 19–23, 25–31]	0.01~0.02 kg
Fuling [10–21, 31]	0.01~0.02 kg
Huangqi [10, 12–16, 18–22, 25, 26, 28, 30–32]	0.01~0.03 kg
Gancao [11–16, 19–23, 25, 26, 30–33]	0.01 kg
Danggui [11, 13–15, 19, 20, 22, 25, 30]	0.01 kg
Chenpi [10, 12, 16, 18, 25, 30, 31]	0.01 kg
Banxia [10, 12, 16, 18, 25, 31]	0.01 kg
Baishao [14, 15, 22, 25]	0.01~0.02 kg
Shanzha [9, 12, 24, 26]	0.01~0.02 kg
Jineijin [14, 26, 29]	0.01~0.02 kg

Chinese medicine for tonifying the kidney	
Gouqi [11, 13–15, 19–22, 25–28, 30, 32, 33]	0.01~0.02 kg
Nvzhenzi [11–15, 17, 21, 22, 26–28, 30–33]	0.01~0.02 kg
Buguzhi [10–12, 14, 17–21, 23, 26–28, 30]	0.01~0.02 kg
Tusizi [10–12, 18–20, 25, 27, 30]	0.01~0.02 kg
Yiyiren [12, 16, 17, 21, 26, 30, 32, 33]	0.02 kg
Ejiao [9, 18, 19, 24, 25, 31]	0.01 kg
Shudihuang [9, 13, 19, 21, 24, 25]	0.01~0.02 kg
Shanzhuyu [13–15, 22, 26]	0.01~0.02 kg
Jixueteng [13, 14, 17, 21, 31]	0.02~0.03 kg
Huangjing [21, 29, 31]	0.01~0.02 kg
